# Transformations of the spatial activity manifold convey aversive information in CA3

**DOI:** 10.1073/pnas.2517639123

**Published:** 2026-06-12

**Authors:** Albert Miguel-López, Negar Nikbahkt, Carlos Wert-Carvajal, Lena Johanna Gschossmann, Martin Pofahl, Heinz Beck, Tatjana Tchumatchenko

**Affiliations:** ^a^ Universität Bonn, Universitätsk-linikum Bonn, Institute for Experimental Epileptology and Cognition Research, Bonn 53127, Germany

**Keywords:** hippocampal circuit, computation, neural activity, spatial coding

## Abstract

Hippocampal circuits form cognitive maps that represent spatial position and integrate contextual information, including affective cues, into episodic memory representations. We investigated how spatial and affective information are combined in the population activity of CA3 axons by analyzing the activity of intermediate-to-dorsal and dorsal-to-dorsal axons in mice navigating a linear track before, during, and after exposure to an aversive air puff stimulus. Both axonal populations maintained a robust, time-invariant activity manifold that encoded spatial information independent of affective context. Deformations of this common manifold encoded the presence of the aversive stimulus without disrupting the spatial representation. Despite differences in spatial coding, both axonal populations encoded affective information with similar efficacy. This population-level encoding was distributed similarly across place and nonplace cells. Our findings demonstrate that hippocampal CA3 axons integrate spatial and affective information within a common representational geometry while maintaining the separability of each information type.

## Significance

The hippocampus helps us remember where we are and how we feel in different situations. We studied how the hippocampus combines location and affective experiences by recording activity from specific hippocampal connections in mice exploring a track before, during, and after experiencing a harmless but unpleasant air puff. We found that spatial information remained stable even when emotional cues were introduced. At the same time, the system flexibly adjusted to represent the aversive experience without disrupting location signals. Both types of hippocampal pathways encoded emotional information equally well, using both spatial and nonspatial neurons. These findings show how the brain maintains reliable maps of space while also integrating emotional experiences.

The hippocampus has been associated with cognitive maps and episodic memory (1, 2). It integrates information streams, such as sensory or contextual cues (3), into sequential representations. A critical aspect of hippocampal function is its role in the simultaneous processing of both spatial and affective information, particularly in natural behaviors like foraging or threat avoidance. While extensive research has characterized specialized cell types within the hippocampus—e.g., place cells (4, 5), object cells (6), reward cells (7) or goal direction cells (8)—a comprehensive understanding of how salient features of the environment influence these neural representations remains incomplete. In particular, the interplay between spatial and affective information during natural behaviors is an important but underexplored area of research. For example, a previous study (9) reported that aversive task representations remained consistent across spatial contexts. Here, we focus on the complementary question of how space representations can remain stable under aversive contextual changes.

We investigated how aversive information alters spatial hippocampal code by analyzing the population activity of long-range projections of CA3 pyramidal cells in a navigation task. We specifically selected CA3 for its rapid spatial information acquisition (10), robust spatial tuning (11), and the role of ventral CA3 neurons in processing aversive information (12, 13). We analyzed the activity of individual CA3 axons projecting from the intermediate to the dorsal hippocampus, as well as commissural CA3 axons originating in the contralateral dorsal hippocampus. In this dataset, an aversive stimulus in the form of an air puff was introduced and later removed. Our analysis focused on how axonal population activity preserved spatial representations over time and integrated affective contextual information in response to the aversive cue’s introduction and removal.

We observed a remarkable consistency in spatial representations across sessions, indicating a robust organizational structure and common task abstraction. Spatial and affective information were not represented separately but were jointly embedded within a unified neural manifold synthesized by the axonal population activity. We observed that intermediate-dorsal axons had greater plasticity in response to aversive conditioning compared to their dorsal recurrent counterparts, although that did not result in a better encoding of aversive information. Notably, removing the aversive stimulus resulted in a distinct poststimulus state rather than returning to prestimulus activity, indicating an affective memory effect. Finally, affective information was embedded across cell types and dimensions, with nonplace cells contributing as much information as place cells to contextual decoding.

Overall, our results indicate that affective information is directly embedded within the hippocampal state-space, deforming its existing manifold to create an integrated representational space while preserving consistent spatial information across time. Our work provides crucial insights into the mechanisms by which the hippocampus creates comprehensive cognitive schemas that incorporate both spatial and affective components of experience.

## Results

**Fig. 1.**
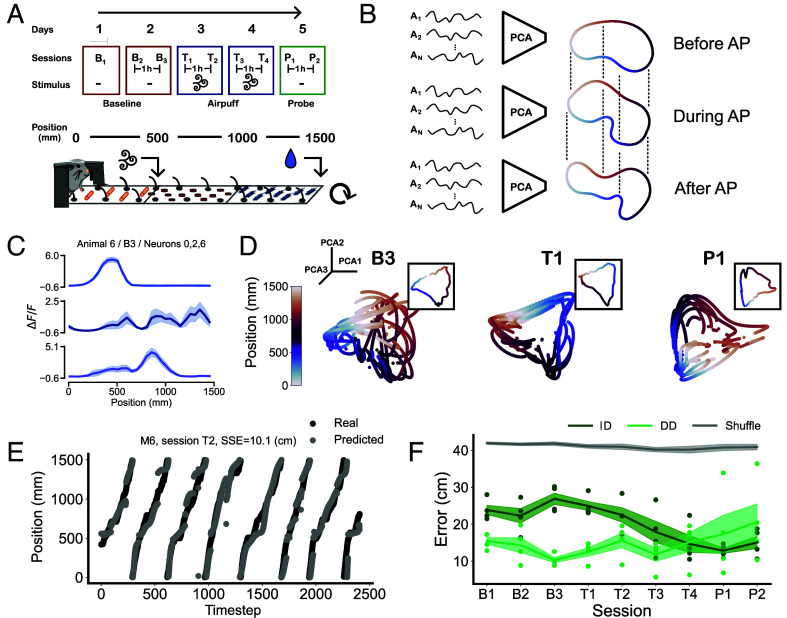
Representation of belt position in ID and DD axons remains robust as manifold position and orientation varies across sessions. (*A*) Sketch of the task design. The *Top* panel represents the behavior protocol. Each box represents a day of recording, and sessions in the same box are separated by only 1 h time interval. T (training) sessions include an aversive stimulus, absent in B (baseline) and P (probe) sessions. The *Bottom* panel shows the behavioral apparatus design, with a head-fixed mouse running on a circular belt. A liquid reward was given at the end of each lap. The aversive stimulus only appears in T sessions at the 500 mm mark. (*B*) A hypothesis of how the manifold shape may change as a function of context changes across baseline, air-puff and probe sessions. The left column represents the axonal activities from each session, reduced to a latent space through PCA. Note that axons may be different from one session to another. Dashed lines indicate how representations of specific positions in space may change over time. (*C*) ΔF/F averaged across trials for an example animal and session, three neurons shown. Shaded areas show the SEM of the data. (*D*) Three-dimensional plots of the latent space for an example animal and three representative sessions. Color represents the position on the belt. *Insets* show the trial-averaged data for that session. (*E*) Position prediction on an example session. (*F*) Prediction performance averaged over mice, for each session and axon type (ID or DD). Scatter dots are the averaged prediction errors for each mouse and session. The gray line is the average prediction error after shuffling the position information (10 repeats per session). Shaded areas show the SEM of the data. ID and DD prediction errors were significantly different than shuffle (Mann–Whitney U test, P<0.05, mice and sessions pooled together), and significantly different from each other.

To understand the integration of spatial and affective information at the population level in CA3, we consider intermediate-to-dorsal (ID) and dorsal-to-dorsal (DD) axonal activity. Our goal is to understand the geometry of population encoding for spatial and aversive information and to clarify how they are distributed along the activity manifold. We analyze a previously published dataset (13) in which mice ran on a 1,500 mm long looping linear belt (Fig. 1*A*) and a reward was delivered at the end of each belt run (trial). Tactile cues allowed the animal to differentiate each third of the belt and estimate its location. In baseline sessions $\left(B_{1-3}\right)$, taking place on the first 2 d, the animals ran across the belt without any external intervention except for the reward. On the third and fourth training days, an air puff (AP) was presented in all trials whenever the animal reached the position x = 500 mm (training sessions, $T_{1-4}$) and was subsequently removed on day 5 (probe sessions, $P_{1-2}$). Population-wide calcium signals were recorded and transformed to dF/F (Fig. 1*C*) from ID axons in 4 mice (M0–M3), and from DD axons in a separate group of 4 mice (M4–M7), under the same behavioral protocol.

Our aim was to understand how an aversive stimulus could reshape the population manifold over time, and how it interacted with the encoding of positional information (Fig. 1*B*). To this end, we characterized the latent space of each recording using principal component analysis (PCA), which reflected the periodic nature of the belt (Fig. 1*D*), indicating that the animals are forming predictive, task-relevant spatial representations. While the exact shape and coordinates of these manifolds changed from session to session, the overall periodic structure was robust across all sessions. This latent space had lower dimensionality than the original, with less than half of the original dimensions being necessary to explain 80% of the variance (*SI Appendix*, Fig. S1*B*–*D*). This indicated that neuronal activity was highly correlated, but not to a large degree. To quantify this activity manifold, we trained a decoder to predict the position of the animal from the latent axonal activity, retaining enough principal components (PCs) to explain 90% of the variance for each session. We were able to predict position accurately (Fig. 1*E*), with the prediction error being significantly different from shuffle controls for all sessions and axonal types (Fig. 1*F*). Task information appeared to accumulate on the first few dimensions, with the error reaching a once enough dimensions were included to explain around 25% of the variance (*SI Appendix*, Fig. S1*E*), suggesting that the spatial code drives a large amount of the correlated activity. Interestingly, ID axons had higher errors during the baseline sessions, which lowered to the level of DD errors after introducing the aversive cue during T sessions. This suggests that the population activity of ID axons improved its spatial encoding as a reaction to the aversive stimulus, while DD kept a more stable spatial representation over sessions.

### Common Spatial Manifolds Exist Across Sessions.

**Fig. 2.**
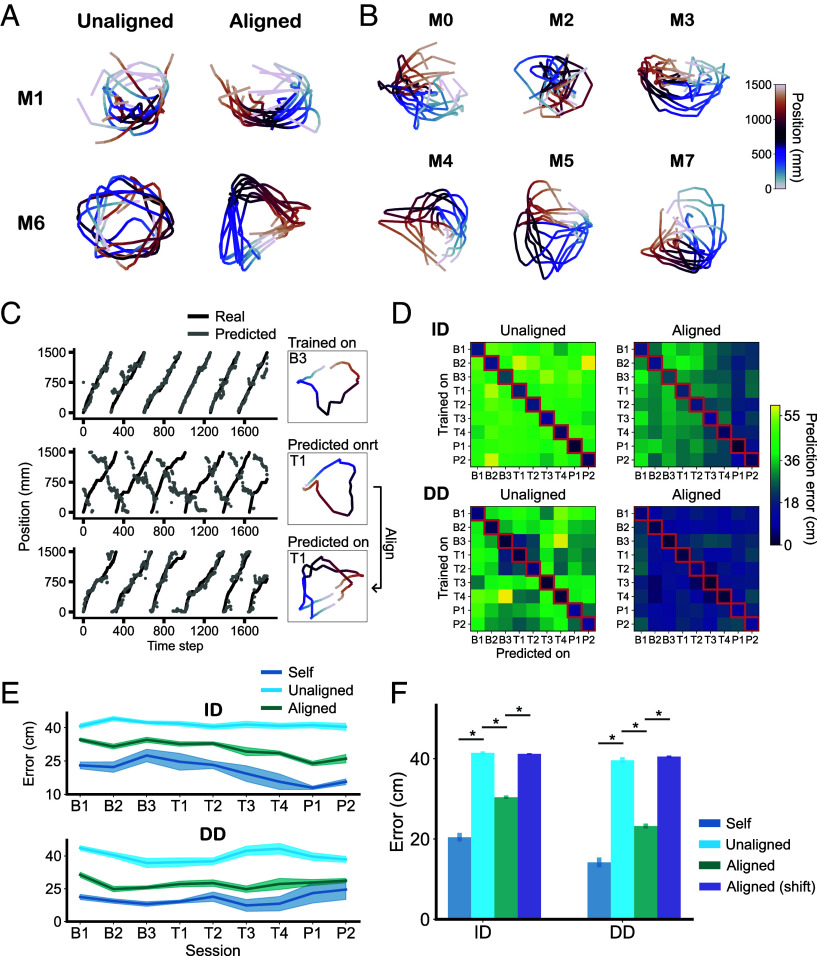
Revealing a common spatial manifold across sessions in axonal population activity. (*A*) Spatial manifolds in ID axons (*Top*) and DD axons (*Bottom* row) can be aligned across task types indicating robust spatial representation space that is independent of task-context. The first three dimensions of session-averaged PCA, before and after alignment, are shown. (*B*) Aligning the population activity across all sessions within individual mice (mice M0, M2, M3, M4, M5, M7) reveals manifold similarities. (*C*) Ground truth position (black) vs. prediction (gray) for a cross-validated $B_{3}$ session (*Top*), for a decoder trained on $B_{3}$ predicting on $T_{1}$ (*Middle*), and predicting on aligned $T_{1}$ data (*Bottom*). Mouse M6 used as an example. The right column shows the session-averaged PCAs. (*D*) Each grid shows all possible combinations of position prediction before (left column) and after (right column) alignment. The first row is the average across ID mice (N = 4), and the second is the average across DD mice (N = 4). Diagonal squares (red border) correspond to cross-validated self-error, while the nondiagonal squares correspond to cross-session prediction. (*E*) Prediction error across sessions for each axonal type. The shaded area represents the SEM of the data. (*F*) Animal averaged errors in ID vs. DD populations. Asterisks indicate a significant difference (Mann–Whitney U test for self to unaligned, paired Wilcoxon test for the others, (P<0.05). Vertical lines show the SEM of the data.

Even though neuronal latent structures appear similar over sessions, a decoder trained on the position of an animal on one session will generally perform poorly when predicting on a different one (Fig. 2*A*, *Middle*, and “unaligned” in Fig. 2*D*). Within-session decoding results showed that the task was generally well represented in each session, suggesting that the mismatch could be caused by similar latent representations occupying different parts of the latent space, or differing from one another by a rotation or other kinds of transformations. Only some axons were shared from session to session, and those can have their stimulus-dependent responses drift over time or even remap, creating differences in the input neuronal activity between sessions and overall changes in the latent space, even if the general ring-like shape remains the same. One could understand the latent space of each session as the projection of an underlying common manifold (14, 15) onto the recorded activity of each day. Following this idea, we expected to find commonalities across sessions under the application of the right transformations.

To test this hypothesis, we aligned the activity manifolds in the principal component space across all sessions using multiset canonical component analysis (mCCA), which maximizes their cross-correlations through linear transformations (*Materials and Methods*). Before alignment, activity manifolds displayed similar periodic structures but in different shapes and angles (Fig. 2*A*, left column). These differences were reduced through the alignment procedure, as seen in the right column of Fig. 2 *A* and *B*. To quantify the strength of the alignment, we assessed spatial encoding stability via cross-session decoder generalization, following the process shown in Fig. 2*C*. To avoid favoring any particular session, we trained and tested a decoder for every possible session pair (Fig. 2*D*). We observed a clear reduction in the cross-session prediction error after alignment (nondiagonal cells in Fig. 2 *D* and *F*). This improvement persisted for the entirety of the experiment (Fig. 2*E*), even for ID axons during the initial sessions, which had a larger within-session error than the rest (Fig. 1*F*). Overall, the error in aligned sessions was significantly lower than in unaligned ones and much closer to the error obtained when predicting the same session (Fig. 2*F*), for both ID and DD axons. To eliminate the possibility that mCCA was able to align datasets independently of their underlying structure, we repeated the process on data that had a random temporal shift applied on each session so that the latent structure became uncorrelated with position but kept its temporal correlations intact (*SI Appendix*, Fig. S2). Prediction error was comparable to shuffle in this case (Fig. 2*F*, aligned shift), suggesting that a shared latent structure with a consistent relationship to task variables is needed for mCCA to work. Overall, the success of the alignment procedure demonstrates that a common space manifold was found across all sessions for both axonal types. Note that these results do not inform us of what the origin of these commonalities is. Given the nature of the task, we could expect any topologically equivalent ring-like structure to be aligned through this procedure. The relevance of these results lies in showing that all sessions have a stable and correct task encoding and that a linear transformation is enough to recover a common representation.

To better understand the conditions for a successful alignment, we generated simulated neuronal data with a Poisson spiking model that used a predefined low-dimensional latent space as its ground truth (*SI Appendix*, Fig. S7). When aligning pairs of generated sessions with similar ring-like latent spaces (*SI Appendix*, Fig. S8*A* and “Easy” in *SI Appendix*, Fig. S8*C*), alignment worked almost perfectly. Interestingly, aligning two sessions whose space representations were different in elaborate, nonlinear ways, but still decodable by our predictors, worked similarly well (the result of aligning a twisted ring and a periodic spiral is shown as “Difficult” in *SI Appendix*, Fig. S8*C*). It is only when the underlying latent space represents a different, incompatible task (such as a double ring, “Incompatible” in *SI Appendix*, Fig. S8) or is overtaken by noise (“Noisy” in *SI Appendix*, Fig. S8) that the alignment procedure fails. This indicates that alignment success in this task depends on having accurate task representations, even if the associated latent spaces significantly differ in nonlinear ways, and hence cannot be attributed to similarities in the underlying neuronal latent spaces beyond being topological rings. Capacity for alignment is also not a simple function of neuronal dimensionality, as shown in *SI Appendix*, Fig. S8*B*. Finally, aligning multiple generated sessions using mCCA performs worse compared to single pairs (see the gap between “self” and “aligned” in *SI Appendix*, Fig. S8*D* “Low noise”), which worsens further when the first few sessions have increased noise (“Early noise” in *SI Appendix*, Fig. S8*D*), which mirrors the DD and ID results from Fig. 2*F*, respectively.

### Affective Information Is Encoded Within the Spatial Activity Manifold.

**Fig. 3.**
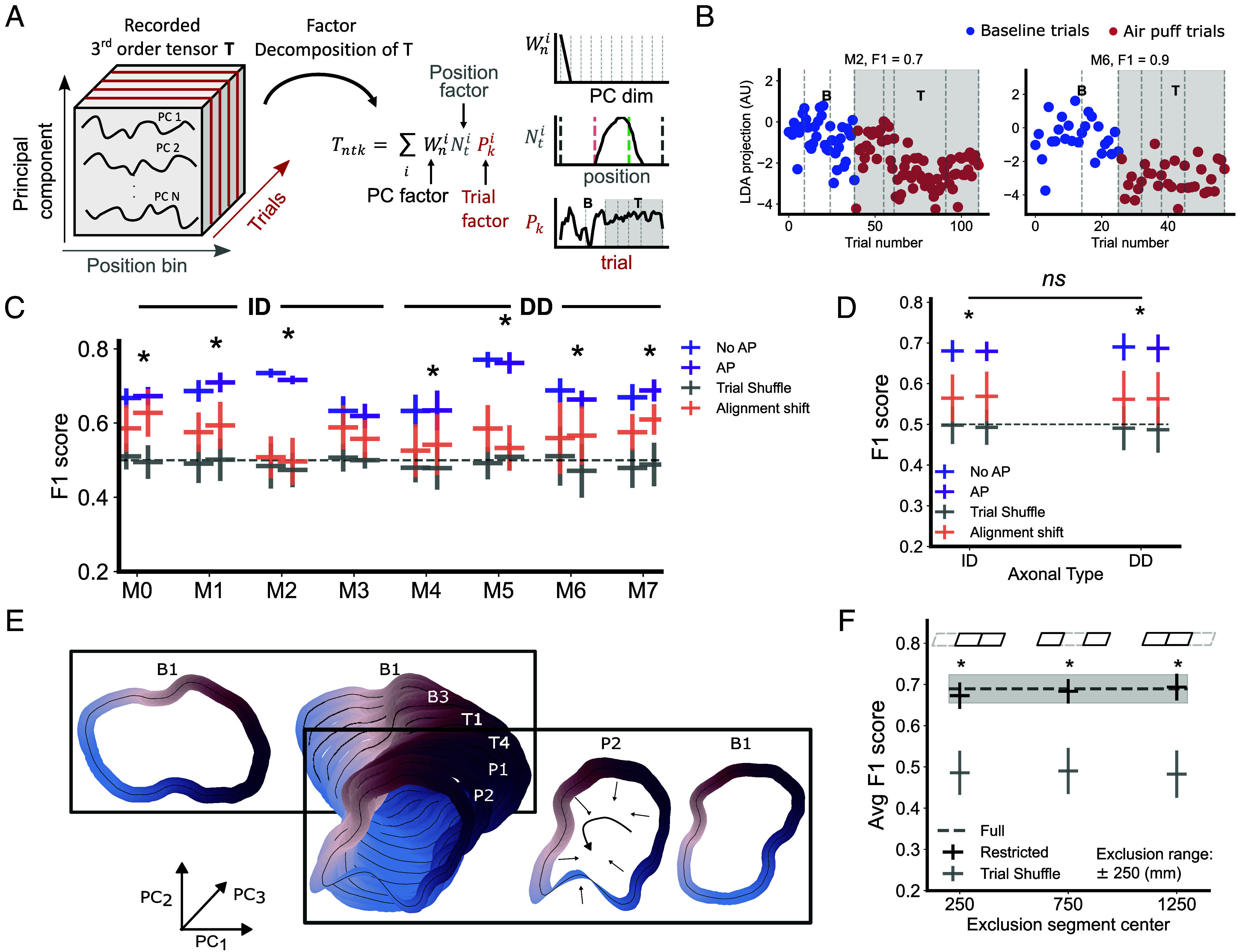
Decomposition of the neural activity tensor identifies dimensions encoding aversive information. (*A*) The recorded 3D tensor (*A*, *Left*) can be decomposed into trial, position, and neural PC factors (*A*, *Middle*). (*A*, *Right*) shows one representative TCA factor for each decomposition. (*B*) 1D projections on the LDA decoding axis. Each dot corresponds to the projection of one trial factor. Session delimiters are marked with dashed lines; air puff sessions have a gray background. The *Left* panel shows mouse M2 (ID axons) and *Right* panel mouse M6 (DD axons). F1 scores are shown on top. (*C*) F1 scores for each mouse and both classes, averaged over TCA repetitions. Vertical lines show the SD of the data. Mice whose F1 scores are significantly (Mann–Whitney U test, P<0.05) above trial shuffle (gray) and alignment shift (brown) controls for both classes are marked with an asterisk. This was further corroborated using two-way ANOVA (*Materials and Methods*). The dashed line indicates the expected F1 score of a completely random guesser. (*D*) Mouse-averaged F1 scores by axon type. Vertical lines show the SD of the data. Asterisks indicate that both classes were above trial shuffle (gray) and alignment shift (brown) controls, as in the previous panel. The difference between ID and DD was nonsignificant (Wilcoxon test, P>0.05. This was further corroborated using two-way ANOVA (*Materials and Methods*). (*E*) Representation of the latent space deformation under contextual changes. Each periodic structure represents the trial-average PCA of a session, with colors indicating position along the periodic belt. As context changes, the latent manifold is modified in ways that keep its space encoding mostly intact. (*F*) Air puff decoding performance when the activity corresponding to certain parts of the belt is excluded. Drawings (*Top*) indicate which third is excluded. The F1 score of the whole dataset (dashed line), as well as the F1 score of the restricted dataset with randomly shuffled labels (gray), are added as a comparison. Vertical lines show the SD of the data. Asterisks denote significance against shuffle (Mann–Whitney U test, P<0.05).

Next, we wanted to understand how the appearance of a novel aversive stimulus (the air puff, or AP) could modify the underlying representational manifold. Since position decoders worked well across all sessions after alignment, we considered the possibility that any contextual changes would happen trial-to-trial rather than timestep-to-timestep. To investigate this hypothesis, we applied tensor component analysis (TCA) to our data (16), a linear technique that decomposes neuronal recordings into three sets of “factors” or components: input dimension-wise, trial bin-wise, and trial-wise. For each mouse, we rearranged our data into a third-order tensor T where the first axis contains the aligned PCs from the previous alignment step, the second axis contains the position-normalized bins for each trial, and the third axis concatenates all trials from baseline to probe sessions (Fig. 3*A*, *Left*). We concatenated sessions using the aligned dimensions because we are interested in observing changes in the common latent space across time and trials. TCA performs dimensionality reduction on the tensor as a whole, decomposing each element of T into the sum of three sets of factors (Fig. 3*A*, see *Materials and Methods*). Intuitively, the process can be understood as performing a PC decomposition with an added third source of variability—i.e., trials—and removing the while removing the orthogonality constraint.

Fig. 3*A*, *Right*, shows the first component of each TCA decomposition for a representative session (more components are shown in *SI Appendix*, Fig. S3*A*). The PC factor shows the weights for each aligned PC dimension, exhibiting a highly selective distribution across the discrete principal components, with some dimensions being fully active while others are not. This shows that TCA is picking up on the dimensions already discovered by PCA or CCA, without further reducing the dimensionality of the data, suggesting that the initial process of dimensionality reduction already found the relevant (linear) components. The position component has a place-cell-like shape, indicating that TCA is merging place field information, likely coming from the PCs. The third dimension exhibits fluctuations across the trial space, indicating how the amplitude of this activity pattern changes across trials. A geometric intuition for these varying trial factors is given in Fig. 3*E*. As components gain and lose strength over trials and sessions, the overall manifold is deformed along selected dimensions, keeping positional information intact (illustrated by the arrows showing $P_{2}$ vs. $B_{1}$ and the preserved periodic shape).

To quantify this variability, we focused on evaluating fluctuations of the trial components across session types. Initially, we solely considered changes accounting for the learning of aversive stimuli, comparing baseline vs. training sessions. We employed linear discriminant analysis (LDA) to decode air puff presence from the trial components using leave-one-out cross-validation (Fig. 3*B*) and evaluated it through the F1 scores for each class. We used scores for randomly shuffled labels as a baseline for comparison (“trial shuffle” in Fig. 3 *C* and *D*). Additionally, we considered the possibility that the decoder was simply picking out session-to-session variability independent of air puff presence. Such variability might arise through a flawed alignment process, changes due to the passage of time such as drift, or the animal becoming more accustomed to the belt environment. To control for this, we repeated the analysis using sessions that were each independently shifted by a random interval, which CCA failed to align successfully (Fig. 2*F*). Air puff decodability was significantly above both control types for all mice (Fig. 3*C*, raw accuracy metrics in *SI Appendix*, Fig. S3 *B* and *C*), indicating that the common manifold changed due to the introduction of an aversive stimulus, rather than session-to-session misalignments or changes unrelated to the air puff. Notably, we found that air puff presence was decodable in both ID and DD axonal recordings, showing no meaningful differences (Fig. 3*D*), indicating that both axonal types react to aversive information similarly. This appears to challenge the idea that ID axons encode more affective information than DD (17, 18).

To rule out that air puff decoding was caused by behavioral correlates—e.g., the animal slowing down in the air puff area – we recomputed the trial factors and repeated the analysis using only activity from particular sections of the belt (*SI Appendix*, Fig. S4*A*). Excluding one-third of the belt did not significantly alter the results, even when the excluded section included the AP or pre-AP belt zones where the animal slows down during T sessions (Fig. 3*F* and *SI Appendix*, Fig. S4*C*). Only one mouse (M2 in *SI Appendix*, Fig. S4*B*) appeared to show a significant reduction in this case. Interestingly, the results remained stable even when two-thirds of the belt were excluded (*SI Appendix*, Fig. S4*D*), suggesting that aversive information is distributed along the entire latent manifold regardless of position. It also shows that the deformations schematized in Fig. 3*A* are pervasive along the belt, not just in locations where the animal modifies its behavior. As an additional test for potential confounders, we applied the air puff decoding procedure to simulated data, in which trials from two types of sessions (characterized by their respective latent space) had to be differentiated after session alignment (*SI Appendix*, Fig. S8*E*). This process returned F1 scores at random guess level When the underlying latent spaces used to generate the data were the same (“Same”), but not when each session class was biased toward different sections of the belt (“Different”). We tested whether or not the class decoders, analogous to air puff decoders in the real data, were simply decoding differences caused by failures in the alignment procedure, by shifting each session by a random amount (“Alignment shift,” same as in Fig. 3*C*). This again resulted in F1 scores at chance level. The same happened when one session class had too much noise to represent the task accurately (“Poor representation”). It is only when the latent spaces of each session type corresponded to clear, correct, but differing representations of the task that we were able to decode trials using this procedure, and found F1 scores comparable to those in the real data. Furthermore, these results did not depend on input dimensionality (representing the number of recorded cells), with the cases other than “Different” remaining at chance level F1 scores regardless of the number of input dimensions (*SI Appendix*, Fig. S8*F*).

### Probe Sessions Constitute a Distinct Postaversive State.

**Fig. 4.**
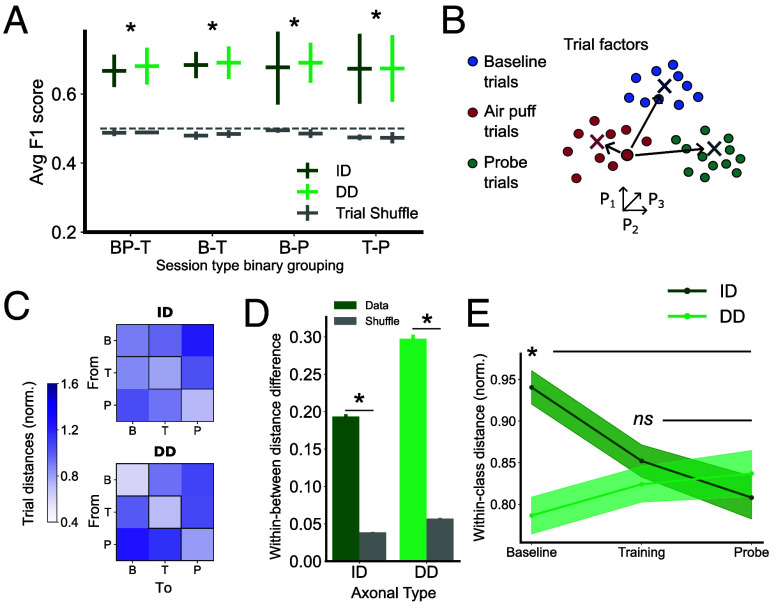
Sessions before, during, and after the air puff consolidated in their own distinct activity patterns. (*A*) F1 scores for different binary comparisons of the three session types, and for each axonal type. “B” are baseline sessions, “T” are air puff sessions, and “P” are probe sessions. The x-axis labels indicate the groups of sessions compared against each other (e.g., in BP-T we distinguish trials belonging to either B or P sessions from T sessions). An * indicates that both axon types for that session grouping were significantly (Mann–Whitney U test, P<0.05) above shuffle (in gray). The dashed line indicates the difference expected from random draws. Vertical lines show the SD of the data. (*B*) Computing distances in trial factor space. Each trial of a session is represented by a single point whose xyz- components are amplitudes of the corresponding trial dimensions. For visualization, we show three trial factor dimensions (x, y, and z axes) and color the activity vectors in blue, red, and green if they correspond to baseline, trial, or probe sessions, respectively. Crosses represent the centroids of each cluster. A single trial is highlighted as an example, with solid black lines indicating the distances from the trial to the centroids. (*C*) Colormap of the average normalized distance from a trial to each centroid, by axonal type. The diagonal elements, highlighted by a black outline, show the average within-cluster distance. (*D*) Average difference between the “within” and “between” normalized distances for each axonal type, for both the original data and the label-shuffled data. Significance (Mann–Whitney U test, P<0.05) was computed with respect to the results from shuffled label data. Vertical lines show the SEM of the data. (*E*) Within class normalized distances across session types for ID and DD axons. Asterisks indicate significance (Mann–Whitney U test, P<0.05) between ID and DD differences relative to the Probe distances. Shaded areas represent the SD of the data.

Next, we extended this analysis to include probe trials. After removing the aversive stimulus, animal behavior became more variable, with stopping behavior spreading out in a wider space interval (13). This made us question whether axonal activity during probe trials significantly differed from baseline and air puff trials. When trying to decode air puff presence by grouping B and P sessions together against T sessions (BP-T in Fig. 4*A*), performance was similar to our previous analysis comparing only B against T. But this was also true when trying to differentiate B from P trials, which lacked the presence of an air puff (B-P in Fig. 3*A*). This indicated that axonal representations in the post-AP context became distinct from the pre-AP context, rather than returning to the baseline after removing the aversive stimulus.

Motivated by this finding, we wanted to understand how the different session types were distributed in trial factor space. We hypothesized that each session type would form its own distinct cluster (Fig. 4*B*) and quantified it by measuring the distance between trials and the centers of mass of each session type (Fig. 4*C*). We found that, on average, within-cluster distances were smaller than between-cluster for all session types when compared to shuffled session type labels (Fig. 4*D* and *SI Appendix*, Fig. S5*F*), which is what we would expect if each session was distributed in its own distinct cluster. This result supports the idea that each session type had its own distinct activity pattern, including B and P sessions which had no air puff presence. We corroborated this by repeating the analysis grouping B and P trials together (no air puff or No AP class) against T (air puff class or AP), finding that the No AP class was more spread out than AP (*SI Appendix*, Fig. S5*I*), implying that we were grouping distinct clusters together. Additionally, separability was better in DD axons compared to ID (Fig. 4*D*), which was likely caused by a higher within-class spread before the air puff introduction (Fig. 4*E*). This result aligns with the position decoding results from Fig. 1*F*, suggesting that ID axonal activity stabilizes after an aversive stimulus appears in the environment. Overall, these results indicate that removing the aversive cue caused changes in the latent manifold that consolidated into a new, distinct contextual encoding, which was different from the context before the air puff was introduced.

### Aversive Information Is Evenly Distributed Across Dimensions and Cell Types.

**Fig. 5.**
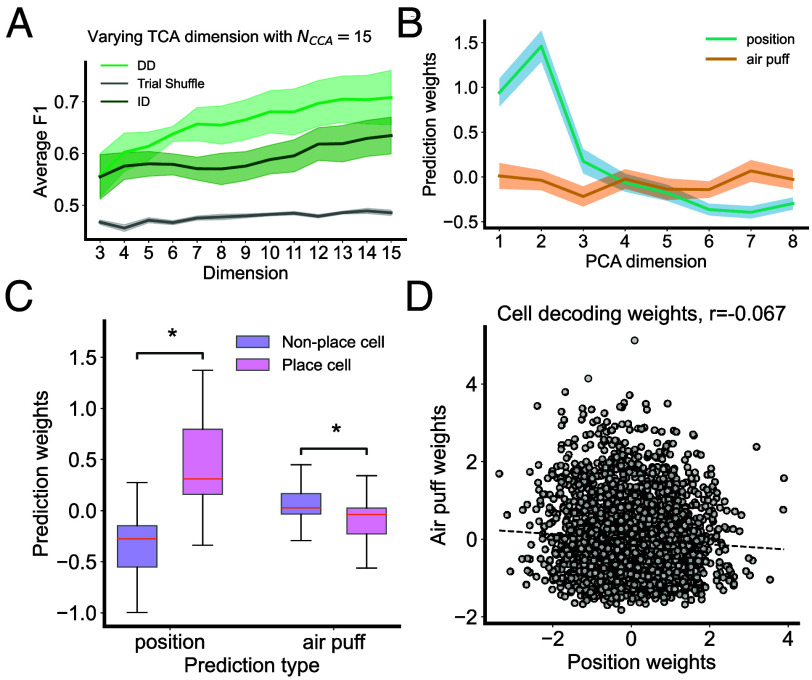
Air puff effect is robust and distributed across cells and dimensions. (*A*) F1 scores plotted against TCA dimensionality, while keeping CCA dimensionality fixed. The shaded area shows the SEM. (*B*) Average prediction weights (see *Materials and Methods* for details) for position and air puff decoding by principal component dimensions. The shaded area shows the SEM. (*C*) Boxplot for the position and air puff decoding weights by cell type. Asterisks denote significance between cell types (Mann–Whitney U test, P<0.05). The colored line within the box represents the median, the box represents the interquartile range (IQR), which includes quartiles 1 to 3, and the whiskers extend 1.5 times outside the IQR. (*D*) Cell weights for air puff decoding plotted against weights for position decoding. Each dot corresponds to a cell. The dotted line shows the linear fit, with a *P*-value of 0.0007.

Finally, we wanted to know how aversive information was encoded across dimensions and axons. Fig. 5*A* shows that the average decodability of the aversive cue increased progressively with the number of TCA dimensions, indicating that aversive information was found beyond just the initial PCs. To understand exactly how affective information is distributed, particularly in comparison to spatial information, we quantified the contribution of each latent dimension or each axon to the decoding process with its decoding weight for either position or air puff decoding (*Materials and Methods*). Dimensionality-wise, initial PCA dimensions contributed the most to position prediction, while air puff information was more evenly distributed along dimensions (Fig. 5*B*), indicating that spatial coding dominates neuronal activity patterns, as they occupy the PCs with larger explained variances. When measuring the contributions of place and nonplace cells (see *Materials and Methods* for classification details), we found, as expected, that place cells had a clear positive contribution to spatial decoding (Fig. 5*C*). But this was not true for air puff decoding, with both cell types contributing a similar amount of information, with even a small but significant difference in favor of nonplace cells. Finally, we found very weak correlation (r=−0.067) between the air puff and position weights of a given axon (Fig. 5*D*), suggesting that aversive information is found in cells independently of their role in position encoding. As comparison, we found a positive correlation (r=0.457) when comparing the position weights obtained from one subset of the data against the other (*SI Appendix*, Fig. S6*F*, see *Materials and Methods* for details). This is consistent with previous reports showing that individual place and nonplace cells contribute significantly to place as well as environmental encoding (19).

To summarize, we have shown that aversive information is similarly distributed between place and nonplace cells independently of their position information, and that nonplace cells have a key role in encoding affective information.

## Discussion

Integrating salient information from the environment into spatial maps is a fundamental cognitive function animals accomplish effortlessly. Yet it remains unclear how this happens at the neuronal level. While changes in single-cell selectivity induced by affective information or task context have been observed across different cortex (20), how the population-wide activity manifolds combine these different information types remains an open question. Previous research has shown that when spatial and nonaffective context variables are simultaneously present during a task, they jointly map into a shared geometric representation (21). Following the idea that hippocampal circuits construct structured maps using the substrate of neural activity manifolds (22–25), it is plausible that existing spatial maps are transformed to accommodate newly arriving information (26). The possibility of a joint encoding of spatial and affective information within a single manifold is further supported by observations that many cell types in the hippocampus can change their firing patterns or acquire novel receptive fields in new task and environmental contexts (13, 27).

We demonstrated that the spatial map generated by a population of CA3 axon populations is preserved across different affective states despite receptive field remapping of individual cells within the population (13, 19). Notably, we show the existence of a common activity manifold with a stable positional representation that remained consistent across days, even after contextual changes caused by the introduction and subsequent removal of an aversive stimulus. The latent representation in the principal component space obtained on each session showed a consistent periodic structure, which reflected the nature of the belt and accurately encoded the position of the animal. However, the specific shape of this spatial representation changed across sessions, preventing a decoder trained on one session from accurately predicting another. These variations may stem from factors such as recording different cell populations, remapping, or representational drift. Crucially, these differences largely disappeared after aligning the recordings. This was also true for ID axonal populations, whose spatial representation improved over time. This suggests that the introduction of an aversive stimulus caused the recruitment of more neurons or the improvement of already existing space-encoding cells to strengthen this shared manifold.

We found that the presence of the air puff could be predicted from the activity changes in the aligned manifold at the single trial level, indicating that affective information was encoded in the neuronal activity. We hypothesized that the aversive cue caused a “deformation” effect on the latent activity manifold, stretching its ring-like shape while preserving relative positional encodings. This was observed regardless of which section of the belt we analyzed, suggesting that contextual information was distributed across the entire manifold, and was independent of behavioral factors, such as the animal stopping at the air puff location. These results were further supported with simulations, which showed a clear, distinct latent space was necessary to decode between trial types with this method, and were not merely picking up on misalignments or other nonmeaningful changes across sessions. Another interesting result was that, even though ID axons are thought to carry affective information into the dorsal region, and in our own data showed a stronger reaction to the introduction of an aversive stimulus, both DD and ID axonal populations had similar air puff decodability. It is possible that both regions encode affective information, but this is only visible at the population level, rather than within single cells. Another possibility is that this information is first transmitted to the dorsal region through intermediate-dorsal projections, and then rapidly integrated in dorsal–dorsal projections. Causal studies such as lesion experiments would be required to fully understand this phenomenon.

Further analysis of the probe sessions revealed that neuronal activity following the removal of the aversive cue was best characterized as a distinct neural state, different from the baseline sessions which also lacked air puff presence. This suggests that not just affective, but also memory-affective information (such as the knowledge that a negative stimulus was present in the past) is represented in this common latent manifold.

Finally, we found that contextual information was distributed evenly along dimensions and cell types. In contrast, spatial information was located mainly along the principal components that explained the most variance, as well as in place cells compared to nonplace cells. This result suggests that contextual changes affect the whole manifold beyond the spatial map and that selectivity among hippocampal axons is highly mixed. The fact that nonplace cells contributed at least as much as place cells to contextual decoding implies that nonplace cells might be key to the integration of contextual information in the hippocampus, and we suggest that their importance in hippocampal neuronal maps could be understated. Further research should study how other contextual information may be integrated within common neuronal manifolds and examine how affective information flows from region to region.

In summary, our results indicate that population-wide spatial representations in the hippocampus might be conserved across time and contexts and that affective information is embedded into the state-space through variations in its underlying population dynamics.

## Materials and Methods

### Data Preprocessing.

Calcium image traces are smoothed using a Gaussian kernel with a size of 25 timesteps, each bin being 100 ms. Timesteps where the animal is stationary are excluded in all analysis unless otherwise specified.

### Dimensionality Reduction.

We used PCA to reduce the dimensionality of the data. For a given session with $N_{a}$ axons and T timesteps, we arrange the inferred firing rate $f_{i}\left(t\right)$ of axon i at time t into the activity matrix F of size $\left(N_{a}\times T\right)$. PCA linearly transforms the data along dimensions in descending order of explained variance

$$\begin{array}{c} X=CF, \end{array}$$

where C is the transformation matrix and X the transformed data such that the first dimension contains the largest explained variance, the second contains the second largest, and so on. We truncated X at N dimensions, which we set as the amount of dimensions needed to explain a cumulative 90% of the variance (unless otherwise stated), such that $X\in R^{N\times T}$.

### Position Decoding.

In order to decode the position of the animal along the belt, we transformed the periodic 1D position values to a 2D unit circle. This allowed us to account for periodicity while having smooth, continuous position data. The angle along the unit circle corresponds to the position along the belt, using the mapping $p\to \left({\hat{p}}_{x},{\hat{p}}_{y}\right):=\left(\sin\left(2\pi p/L\right),\cos\left(2\pi p/L\right)\right)$ where p∈[0,1,500) is the current position in millimeters and L=1,500 the length of the belt. Decoding was performed using the package developed by the Kording Lab (28), specifically the Wiener, support vector regression (SVR), and extreme gradient boost (XGBoost) decoders. The error is taken as the average squared error (SSE), which calculates the average squared difference between the prediction and the real position, accounting for periodicity. In any situation where the decoder was trained and tested in the same dataset, we used fivefold cross-validation.

### mCCA.

In order to align the data from different sessions we used a generalization of CCA, a classical statistical method that finds a linear transformation that maximizes the cross-correlation between two datasets. Here, we adapted the algorithmic definition of a mCCA from Via et al. (29) which uses the maximum variance (MAXVAR) definition given by Kettenring (30), where the target is to align the maximum variance axes across datasets.

Let M be the number of datasets and $X^{\left(i\right)}$ the data matrix for dataset i, of size N×T. Our goal is to find a set of linear transformation matrices $H^{\left(i\right)}$ such that the sum of cross-correlations between all possible transformed dataset pairs is maximized. The space of maximum correlation between all datasets is called the “canonical” space. Let $h^{\left(i\right)}$ be the basis vector of the $H^{\left(i\right)}$ transformation matrix, such that $z^{\left(i\right)}=X^{\left(i\right)}h^{\left(i\right)}$ is the projection of dataset i onto the canonical dimension of maximum summed correlation. Our optimization problem reads:

[1] $$\begin{array}{cc} \text{arg}\;{\text{max}}_{h_{1},...,h_{M}}\rho & =\frac{1}{M(M-1)}\sum_{k,l=1;k\neq l}^{M}{z^{\left(k\right)}}^{T}z^{\left(l\right)}\\ & =\frac{1}{M(M-1)}\sum_{k,l=1;k\neq l}^{M}{h^{\left(k\right)}}^{T}R_{\mathrm{kl}}h^{\left(l\right)}, \end{array}$$

$$\begin{array}{c} \text{subject to}\frac{1}{M}\sum_{k=1}^{M}{h^{\left(k\right)}}^{T}R_{\mathrm{kk}}h^{\left(k\right)}=1, \end{array}$$

where $R_{\mathrm{kl}}=X^{\left(k\right)}{X^{\left(k\right)}}^{T}$ is the correlation matrix between two given datasets.

By vertically concatenating all the $h^{\left(i\right)}$ vectors into the vector h of length M×N, and rearranging terms, this optimization problem reduces to the following equation:

$$\frac{1}{M-1}\left(R-G\right)h=\rho Gh,$$

where R is a matrix of size (M∗N)×(M∗N) containing all the $R_{\mathrm{kl}}$ correlation matrices, and G only the autocorrelations in its diagonal. This equation has the well-known form of a generalized eigenvalue problem, defined as Av=λBv, which can be solved algorithmically. In our case, $A=\frac{1}{M-1}\left(R-G\right)$, and B=G.

We solve this system numerically using the *eigh* function from scikit-learn (31). The obtained eigenvectors h correspond to a concatenation of the transformation vectors $h^{\left(k\right)}$ for each dataset. The first eigenvector transforms the data to the canonical dimension with most correlation sum, the second to the dimension of second most correlation sum, and so on. Arranging the $h^{\left(k\right)}$ vectors from each eigenvector in rows gives us the transformation matrix $H^{\left(k\right)}$ for dataset k to the canonical space.

This mCCA solution required all datasets to be of the same size. If the number of latent dimensions in each dataset is $N^{\left(i\right)}$, set to explain at least 90% of the variance (details in *Dimensionality Reduction*), we truncated it at $N_{\mathrm{CCA}}=\;\max_{i=1,...,M}N_{i}$ so that all datasets contained at least that much variance explained. We also needed to equalize the temporal dimension $T^{\left(i\right)}$, as each session has different number of trials, and each trial different number of timesteps (since the time the animal took to complete a run varied). First we warped each trial to have the same number of bins B, set to 150, by averaging the values within each bin. Then we truncated to the smallest number of trials $I_{\mathrm{trunc}}$ across sessions, so that each dataset had the common size $N_{\mathrm{CCA}}\times \left(B*I_{\mathrm{trunc}}\right)$.

These truncated and warped arrays were only used to train the mCCA algorithm, from which we obtained the transformation matrices $H^{\left(i\right)}$. We quantified alignment performance using the full, unwarped datasets. There exist M+1 linear spaces where the data can be aligned: the canonical space and each of the M starting spaces. To align to the space of a dataset k, we transformed all other $X_{i\neq k}^{\left(i\right)}$ to the canonical space, and then backtransformed to the k-th space using ${H^{\left(k\right)}}^{-1}={H^{\left(k\right)}}^{T}$. We chose the alignment space with the highest average cross-session position prediction. Each value was obtained by training a position decoder on one aligned dataset and testing on another. There are M self-predictions (tested on the training dataset) and M(M−1) cross-predictions, done in both unaligned and aligned data. When predicting on the same session (self-prediction) fivefold cross-validation was used.

Additionally, we added a “shifted” control that tries to align datasets that have been each temporally shifted by different random amounts, ensuring that the relationships between population activity and position are broken while internal temporal correlations remain intact. The results are averaged across 20 random combinations of shifts for each animal.

#### TCA.

We performed a tensor decomposition analysis using our own notation of PC factors, position factors, and trial factors adapted from Williams et al. (16), which was originally introduced using neuron, time, and trial factors. Note that dimensionality reduction using TCA generally decomposes a third-order tensor N × T × K to a reduced representation with R≤N dimensions, each having two associated vectors of T and K weights, respectively. N represents the dimensionality of the input data, which corresponds to neurons in raw data, and Principal Components in our aligned datasets. T represents the temporal evolution during a trial, usually the number of time bins. Since in our task the animal traverses in a single direction, the temporal evolution has a 1 to 1 relationship with the position evolution. Our data are normalized using position bins, rather than time bins (which are beholden to the animal’s velocity during a trial, so that, e.g., half the belt might not always be traversed in half of the trial time), which we use to represent the temporal evolution during a trial. The number of TCA dimensions was set to be the same as the number of CCA dimensions, selected to explain at least 90% of the variance on all datasets. We set the number of replicates to 10 and selected the non-negative Parafac decomposition method with hierarchical alternative least squares(*ncp hals*). The optimization algorithm is not guaranteed to find a global minimum, and often multiple similar solutions are found for the same data. We repeated the analysis at least 10 times for each quantification and computed their average. Let us note that the differences for each repeat were small and did not alter the overall result.

To create the input for TCA, we truncated the aligned data at the common latent dimension and each trial normalized to have the same length (see previous section for details). Then the trials from all sessions were concatenated into a 3D tensor T of size $N_{\mathrm{CCA}}\times I\times B$, whereI is the total number of trials for that animal, and fed into the TCA. As a result we obtained three matrices: the dimensional factors D of size $N_{\mathrm{CCA}}\times N_{\mathrm{TCA}}$, the trial factors S of size $I\times N_{\mathrm{TCA}}$, and the temporal factors E of size $B\times N_{\mathrm{TCA}}$.

### Air Puff Decoding.

To decode the air puff presence in a given trial, we applied the LDA algorithm as included in the python package scikit-learn (31) on the trial factors. We chose the eigenvalue decomposition method with no shrinkage factor. Since it is a binary classification task (two labels), we projected the data onto a 1D axis. The F1 results were computed using leave-one-out cross-validation. Additionally, to avoid data imbalance issues, multiple repeats of the same decoding were performed, choosing random subsets of the trials such that both classes always had the same number of samples. This was repeated 10 times with different trial subsets.

For the label shuffle control, class labels were randomly scrambled before training the LDA decoder, averaging the results over 10 random shuffles. We used the trial factors from the best F1 attempt in the previous step. For the “align (shift)” control, we used the data resulting from the random shifts as described in *mCCA*.

We tested significance in two ways. The first involved testing significance against every control and for each class individually, requiring all of them to be significant. We computed the *P*-values using the nonparametric Mann–Whitney U test between the F1 scores and the two controls (“trial shuffle” and “aligned (shift)”). When testing mice individually or in groups (Fig. 3 *C* and *D*), this was done for both decoding classes (“AP” and “No AP”). When testing session groupings (Fig. 4*A*), this was done for both axonal types (“ID” or “DD”). This returned 4 *P*-values for each test, and we required all to be below 0.05 to accept the F1 scores as significant with respect to the controls (indicated with asterisks).

The second method we used was two-way ANOVA. The measurement was F1 scores, the first independent variable was the “analysis type” (normal, trial shuffle, or shifted alignment), and the second independent variable was either the “decoding class” (AP or No AP, Fig. 3) or the “axonal types” (ID or DD), Figs. 3*D* and 4*A*). The *P*-values for the influence of analysis type was under 0.05 in all cases, corroborating that the obtained F1 scores differ from controls. *P*-values for “axonal types” were nonsignificant, as well as “decoding class.”

To exclude part of the belt in other control analysis, we defined an exclusion position interval and eliminated the corresponding bins from the tensor T.

### Trial Distances.

For the trial distance analysis, we computed Euclidean distances between each trial factor, corresponding to the rows in matrix S, and the center of mass of each class (defined by either session type, or the presence of an air puff). For these calculations, we set $N_{\mathrm{TCA}}$ to be 11 for all mice, as to compare distances in the same N-dimensional space. We chose this value as the average across mice to explain 90% of the variance.

For the within-cluster distance significances, we first calculated the difference between either B or T distances to P, and then did significance testing on the ID group difference against the DD group difference.

#### Decoding weights.

To quantify the contribution of a feature (axon or latent dimension) to a given decoding, we used the weights from linear decoders for position and air puff decoding.

#### Position weights.

For position, we used the coefficients obtained from Wiener filter regression, which for the predicted position $\tilde{p}=\left({\tilde{p}}_{x},{\tilde{p}}_{y}\right)\approx \left({\hat{p}}_{x},{\hat{p}}_{y}\right)$ are given by the weights ω in

$$\begin{array}{c} {\tilde{p}}^{\left(k\right)}\left(t\right)=\sum_{i=1}^{N}\omega_{i}^{k}x_{i}\left(t\right), \end{array}$$

where k=x or y (one of the two coordinates after the 2D transformation), $x_{i}\left(t\right)$ is the value of feature i at time t (in our case, the PCA dimensions), and N is the total number of features considered in the analysis (see *Position Decoding* for details). We took the contribution of PCA dimension i to position decoding as the average $\omega_{i}=\left(\left|\omega_{i}^{x}\right|+\left|\omega_{i}^{y}\right|\right)/2$.

Given the PCA transformation matrix C (*Dimensionality Reduction*), its elements $c_{\mathrm{ij}}$ denote the contribution of cell $j=1,\ldots ,N_{a}$ to principal component i. And conversely, since C is orthogonal, $c_{\mathrm{ji}}$ is the loading of dimension i onto cell j, such that its reconstructed firing rate is given by $f_{j}\left(t\right)=\sum_{i}c_{\mathrm{ji}}x_{i}\left(t\right)$. We then defined the contribution of cell j to position decoding as

$$\begin{array}{c} \omega_{j}^{p}=\sum_{j=1}^{N}\left|c_{\mathrm{ji}}\right|\omega_{i}. \end{array}$$

Intuitively, if $\left|c_{\mathrm{ji}}\right|$ is relatively large, cell j is a major contributor to variations in principal component i, and hence a main contributor to decoding weight $\omega_{i}$.

Finally, weights $\omega_{j}^{p}$ were z-scored to allow comparison between different sessions and animals.

As a control, we calculated the position weights obtained from the same cells when computed from different subsets of the data, in order to estimate the correlation. Each dataset was split in two, one containing the first two-thirds of the data and the second containing the last two-thirds (allowing for one-third of overlap). The decoding weight calculation was done as detailed above for both datasets, and then plotted against each other.

#### Air puff weights.

Air puff presence was decoded using LDA (*Air Puff Decoding*). Its input are the TCA trial factors corresponding to the rows $S_{i}$ in matrix S (*TCA*), each of length $N_{\mathrm{TCA}}$. Since our classification problem is binary, LDA projects the data to a 1D space using the vectors $\omega^{\mathrm{LDA}}$:

$$\begin{array}{c} LDA_{i}^{\mathrm{proj}}=\sum_{j=1}^{N_{\mathrm{TCA}}}\omega_{j}^{\mathrm{LDA}}s_{\mathrm{ij}}, \end{array}$$

where $\omega_{i}^{\mathrm{LDA}}$ is the LDA weight for TCA dimension j and $s_{\mathrm{ij}}$ the element ij from S. The weights were averaged over cross-validation folds and data balancing repetitions, to obtain a final $\omega^{\mathrm{LDA}}$ weight vector.

Next, we computed the decoding contribution of each CCA aligned dimension using the dimensional factors D, each element $d_{\mathrm{ij}}$ being the loading of CCA dimension i to TCA dimension j, with the formula:

$$\begin{array}{c} \omega_{i}^{\mathrm{CCA}}=\sum_{j=1}^{N_{\mathrm{TCA}}}\left|\omega_{j}^{\mathrm{LDA}}\right|d_{\mathrm{ij}}. \end{array}$$

Obtained weights were averaged over TCA repetitions.

Having the contribution of each CCA dimension, we used the transformation matrices $H^{\left(k\right)}$ to estimate the contribution of each PCA dimension i from session k to air puff decoding with the formula:

$$\begin{array}{c} \omega_{i}^{\mathrm{PCA}}=\sum_{j=1}^{N_{\mathrm{CCA}}}\omega_{j}^{\mathrm{CCA}}H_{\mathrm{ji}}^{\left(k\right)}, \end{array}$$

where we used the backtransformation matrices ${H^{\left(k\right)}}^{-1}={H^{\left(k\right)}}^{T}$

Then, to obtain the contribution of each cell to air puff decoding, we back-transformed from PCA space to axonal space, again using the inverse $C^{-1}=C^{T}$:

$$\begin{array}{c} \omega_{i}^{\mathrm{AP}}=\sum_{j=1}^{N_{\mathrm{PCA}}}\omega_{j}^{\mathrm{PCA}}c_{\mathrm{ji}}. \end{array}$$

To summarize, the contribution to air puff decoding can be reduced to the following matrix multiplication:

$$\begin{array}{c} \omega^{\mathrm{AP}}=C^{T}{H^{\left(k\right)}}^{T}D\left|\omega^{\mathrm{LDA}}\right|, \end{array}$$

where |·| represents applying the absolute operator element-wise. As with the position weights, the air puff weights $\omega_{i}^{\mathrm{AP}}$ were z-scored to allow comparison between different sessions and animals. Place and nonplace cell classification was defined using the Dombeck standard (32).

## Supplementary Material

Appendix 01 (PDF)

## Data Availability

All experimental data used in this study have been previously published (13) and their online source links are incorporated into our analysis code which is publicly available (https://github.com/amiguello/aversive_analysis_2025.git). DOI will be generated upon acceptance. All other data are included in the manuscript and/or *SI Appendix*.
