# Supplementary material for: Transformations of the spatial activity manifold convey aversive information in CA3
Source: Proc Natl Acad Sci U S A. 2026 Jun 12;123(24):e2517639123. doi: 10.1073/pnas.2517639123 (PMC13273363; doi:10.1073/pnas.2517639123)
Supplement: Supplementary file 1 — Appendix 01 (PDF) [file pnas.2517639123.sapp.pdf]

PNAS

Supporting Information for

Transformations of the spatial activity manifold convey aversive information in CA3

Albert Miguel-López, Negar Nikbahkt, Carlos Wert-Carvajal, Lena Johanna Gschossmann, Martin Pofahl, Heinz Beck, and Tatjana Tchumatchenko

Albert Miguel-López, Heinz Beck, Tatjana Tchumatchenko.
E-mails: albert.miguel@research.fchampalimaud.org, heinz.beck@ukbonn.de, tatjana.tchumatchenko@uni-bonn.de

This PDF file includes:

Supporting text
Figs. S1 to S8

Supporting Information Text

Simulated data. We applied previously described methods on simulated data in order to understand their limitations and address potential concerns. This generated data is not meant to be an accurate reproduction of the real dataset, but rather provide a set of edge cases to probe our methods and test different hypothesis.

Generating positions. First we needed to simulate movement in a periodic 1D space, akin to the real animals moving along the belt. We computed the average velocity and standard deviation at each position in the real data (using only the baseline sessions), and used a normal distribution with the same average and standard deviation to generate velocities at each position. To generate the positions of a full simulated session, we start at the position $p = 0$, draw a velocity value v from the velocity generator, and advance the position by $v * dt$, where $dt = 0.01$ seconds. At the new position, we draw a new velocity value, and continue the process. A "trial" is counted once the position passes 1500, at which point it loops back to 0. The session finishes when a specific number of trials are completed, which are set to 50 unless specified otherwise.

To diminish similarity between trials, random stopping behavior is added to the simulation that reduces the overall velocity in given segments of the belt. Each trial is randomly given between 0 and 3 stops, in positions (p_{stop}) uniformly distributed along the belt. The length of these stops in distance units (p_{length}) is drawn from a uniform distribution between 0 and 375 (one fourth of the real belt), each applying a multiplier v_{mi} to the velocity of the form:

$$\frac{1}{v_{mi}} = \frac{5}{\text{stops}} \exp\left(-10\,\frac{(p - p_{stop})^2}{2{p_{length}}^2}\right)$$

This results in an inverted gaussian multiplying the velocity around stop positions. The actual velocity v_f used to advance the position is:

$$v_f = v \sum_i^{\text{stops}} v_{mi}$$

Neuronal ground truth. To generate realistic spike trains, we defined a set of latent spaces that would serve as the ground truth for each simulation. All these latent spaces are low dimensional and encode movement along a one-dimensional periodic space. Below we describe the ones used in this manuscript.

Modified rings Three-dimensional periodic rings with non-linear alterations. The X and Y dimensions perform a full circle, with amplitudes separately modified through gaussian functions. The Z dimension goes through two cycles, with similarly modified amplitudes. Two variants of the rings were used, defined at each position p in the following way:

$$\begin{aligned}
x(p) &= \cos\left(2\pi p/1500\right)\left(1 + \exp\left(-\frac{(t-750)^2}{2\,(350)^2}\right)\right)\\
y(p) &= \sin\left(2\pi p/1500\right)\left(1 + \exp\left(-\frac{(t-600)^2}{2\,(200)^2}\right)\right)\\
z(p) &= \left(\cos\left(2\pi p/1500\right)\right)^2 \cdot 0.5 \cdot \left(1 + \exp\left(-\frac{(t-750)^2}{2\,(400)^2}\right)\right)
\end{aligned}$$

and

$$\begin{aligned}
x(p) &= \cos\left(2\pi p/1500\right)\left(1 + \exp\left(-\frac{(t-900)^2}{2\,(200)^2}\right)\right)\\
y(p) &= -\sin\left(2\pi p/1500\right) \cdot 2 \cdot \left(1 + \exp\left(-\frac{(t-750)^2}{2\,(350)^2}\right)\right)\\
z(p) &= -\,3 \cdot \left(\cos\left(2\pi p/1500\right)\right)^2
\end{aligned}$$

Note that the purpose of the alterations is simply to prevent a trivial perfect circle to be the ground truth. Each variant changes the direction and amplitude of the deformations.

Rings with sigmoid position Simple circular rings whose input is a sigmoid function of the current position p. This makes the latent space more biased towards a specific segments of the one-dimensional periodic space. We used two variants, one biased towards the start of the virtual belt and another towards the end, defined in the following way:

$$\begin{aligned}
\hat{p} &= \frac{1500}{1 + \exp\left(-\left(p - p_c\right)/p_w\right)}\\
x(\hat{p}) &= \cos\left(2\pi p/1500\right)\\
y(\hat{p}) &= -\sin\left(2\pi p/1500\right)\\
z(\hat{p}) &= 0
\end{aligned}$$

Where $p_c = 375$ was the sigmoid center for one variant, $p_c = 1125$ for the other, and $p_w = 188$ as the sigmoid width for both.

Twisted circle This latent space corresponds to a perfect circle being rotated at each position to imitate a twisted ring, with the height (Z dimension) being tuned so that the shape does not cross itself and accurate space representation can be maintained. For each position p we defined the following horizontal vector $\vec{p} = [x, y, z]$:

$$\begin{aligned} \theta =& 2\pi p/1500 \\ x =& \cos\theta \\ y =& \sin\theta \\ z =& \cos\theta \end{aligned}$$

As well as the rotation matrix R_x around the X axis:

$$R_x = \begin{bmatrix} 1 & 0 & 0 \\ 0 & \cos\theta & -\sin\theta \\ 0 & \sin\theta & \cos\theta \end{bmatrix}$$

The resulting "twisted" circle is then given by the matrix multiplication $\vec{p_t} = [x_t, y_t, z_t] = \vec{p}R_x$. Finally, to prevent this latent space from crossing itself, we add the following amount z_{t+} to the Z axis of p_t:

$$z_{t+} = 0.25\ \exp\left(-\frac{z_t - 375}{2(200)^2}\right)$$

The final result is similar to an "infinity" shape.

Periodic spiral This shape is meant to be a complicated but still valid representation of a periodic one-dimensional space. Each half of the simulated belt corresponds to a circle in the latent space, with radius and height changed to prevent a path through the two circles from touching itself. The first half can be understood as a non-linearly rising spiral, the non-linearity added to prevent the rising axis to be linearly related to the position p:

$$\begin{aligned} x(\hat{p}) =& \cos(4\pi p/1500) \\ y(\hat{p}) =& \sin(4\pi p/1500) \\ z(\hat{p}) =& \frac{1}{1 + \exp(-0.03\,(p - 560))} \end{aligned}$$

for $p \in [0, 750)$. The second half is a descending spiral that starts where the other left off, and finishes at the starting point. Its radius envelops the previous spiral, preventing self-crossings.

$$\begin{aligned} r(p) =& 1 + 2\ \exp\left(-\frac{(p - 1125)^2}{2(100)^2}\right) \\ x(\hat{p}) =& r(p)\ \cos(4\pi p/1500) \\ y(\hat{p}) =& r(p)\ \sin(4\pi p/1500) \\ z(\hat{p}) =& \frac{1}{1 + \exp(-0.025\,(p - 938))} \end{aligned}$$

For $p \in [0, 750)$.

Double ring A simple double lap, which simulates a different task where the animal crosses each position twice. Accurate position prediction is not possible, as each location in the latent space corresponds to two possible positions along the simulated belt.

$$\begin{aligned} x(\hat{p}) =& \cos(4\pi p/1500) \\ y(\hat{p}) =& \sin(4\pi p/1500) \\ z(\hat{p}) =& 0 \end{aligned}$$

Generating spike trains. Neuronal firing rates r were computed as random linear combinations of the three dimensions from a chosen ground truth latent space:

$$r(p) = \omega_1 x(p) + \omega_2 y(p) + \omega_3 z(p)$$

where x, y, and z are the values of the latent space at a given position p as defined in the previous section, and ω_i are random weights chosen from a normal distribution with zero mean and a standard deviation of one. The default amount of neurons per session was 50.

After obtaining the firing rates, we generated spikes following an inhomogeneous Poisson model, so that at each time bin the probability of a spike was given by:

$$P(\text{spike}) = (r(p) + \epsilon(t))\ dt$$

Where $\epsilon(t)$ is the noise, chosen at each time step from a normal distribution with zero mean and a standard deviation of one unless otherwise specified, and dt is the time bin length, the same used for the position generation. Finally, the spike trains are convolved with a gaussian window of length 0.1s to simulate the continuous amplitudes from calcium imaging.

Simulated data processing. Sessions of simulated data were generated using the previous steps, resulting in activity matrices of size $N_a \times T$ with a corresponding position vector, where N_a represents the number of neurons. All the methods of analysis previously described were applied on the simulated data the same way they were on real data. We used support vector regression (SVR) for position decoding. Each value quantification shown in Fig. S8 was calculated using 25 samples. This means 25 pairs of sessions for session-pair, 25 sets of multiple sessions for multi-session alignment, and 25 sets of two-class sessions for class type decoding (the equivalent of air puff decoding).

Aligning generated data Various kinds of alignment are shown in Fig. S8 which require a description. When aligning pairs of sessions, classic pair-wise CCA was applied. In Fig. S8C, each pair type corresponds to a different combination of ground truth latent spaces: "Easy" aligned the two variants of "modified rings", "Different" aligned a "twisted circle" with a "periodic spiras", "Incompatible" aligned a "modified ring" with a "double ring", and "Noisy" aligned a "modified ring" with completely random latent space. For Fig. S8B, 9 simulated sessions were aligned using mCCA, each with 40 neurons and 20 trials. These numbers were chosen to recreate the conditions of analysis of the real data, and the panel is analogous to the ID/DD comparison in Fig. 2F. The first "modified ring" variant was used as the ground truth for sessions 1-3 and 8-9, and the second variant for sessions 4-7. "Early noise" increased the default standard deviation of the firing rate noise to 10 for the first 3 sessions and 5 for the 4th session, the rest remaining at the default of 1. "Low noise" kept it at 1.

Alignment success was evaluated through "alignment performance", which quantifies how close the prediction error after alignment is to the "self" decoding, relative to the unaligned error. Given two datasets 1 and 2, alignment performance for dataset 1 is given by:

$$\text{Alignment performance} = 1 - \frac{SSE_{21}^{\text{aligned}} - SSE_{11}}{SSE_{21}^{\text{unaligned}} - SSE_{11}}$$

Where SSE_{ij} is the averaged squared error for a predictor trained in session i and evaluated on session j, either before or after alignment. If $i = j$, no alignment is needed, and the cross-validated within-session error is given. A value close to 0 means that the error after alignment did not improve relative to pre-alignment, and a value close to 1 indicates that the cross-session decoder worked just as well as the cross-validated within-session decoder.

Session type decoding in generated data Air puff decoding was applied in simulated data just as it was in real data, by computing trial factors using TCA and predicting trial types using LDA. The label to be predicted was generalized to two arbitrary "session classes", with 3 sessions of each kind being generated, all 6 together aligned, and finally predicted on a trial-by-trial basis using the TCA trial factors. Trial type was evaluated through F1 scores like in Fig. 3. For the "Same" category in Fig. S8E, both session types had the same "modified ring" variant as ground truth. In the "Different" category, each class had a different "ring with sigmoid position" variant assigned, so that they would display bias towards different segments of the belt. In "Alignment shift", we used the "Different" ground truths but caused mCCA to fail alignment by warping each session by a random amount, same as the "alignment shift" control in Fig. 3C. "Poor representation" had the "Different" ground truths with noise standard deviations of 25. Once again, each session had 40 neurons and 20 trials.

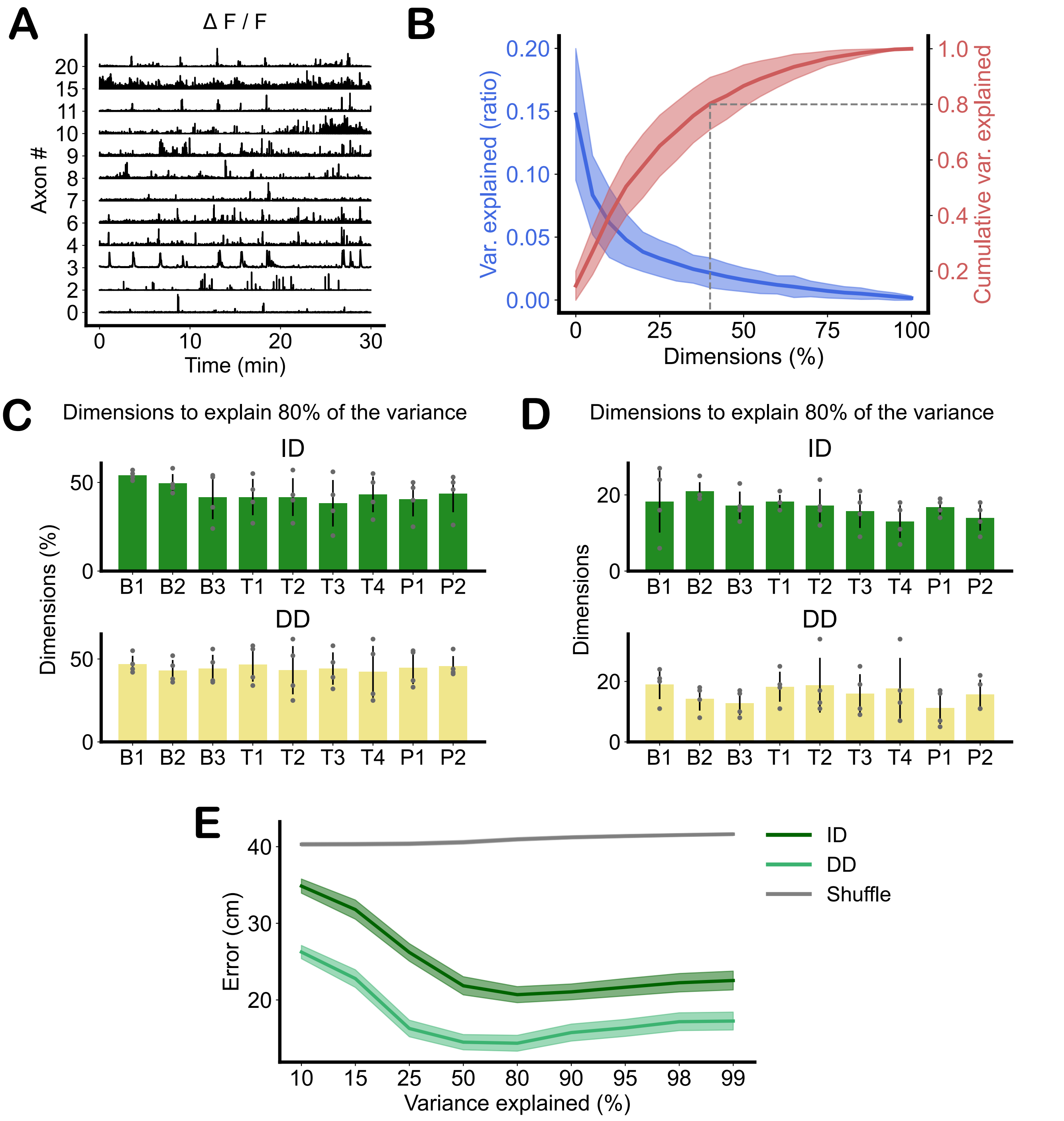

Fig. S1. Raw data and dimensionality plots. A, Example of a $\Delta F/F$ fluorescent signal recorded during a session. **B,** Variance ratio and cumulative variance explained by dimension, averaged over all mice and sessions. Shaded area represents the standard deviation of the data. Dashed line shows the average percentage of dimensions needed to explain 80% of the variance. **C,** Average percentage of dimensions needed to explain 80% of the variance for each session and axon type. Error bar represents the standard deviation. Results are averaged over 4 mice for each, shown as gray dots. Overall we needed $(44\% \pm 10\%$(SD)$)$ to explain 80% of the variance. **D,** Same as in the previous panel but considering the absolute number of dimensions instead of a given percentage. **E,** Error prediction as a function of the variance explained by the latent dimensions. Shown as the average across mice and sessions, with the shaded area being the standard error of the mean (SEM) of the data.

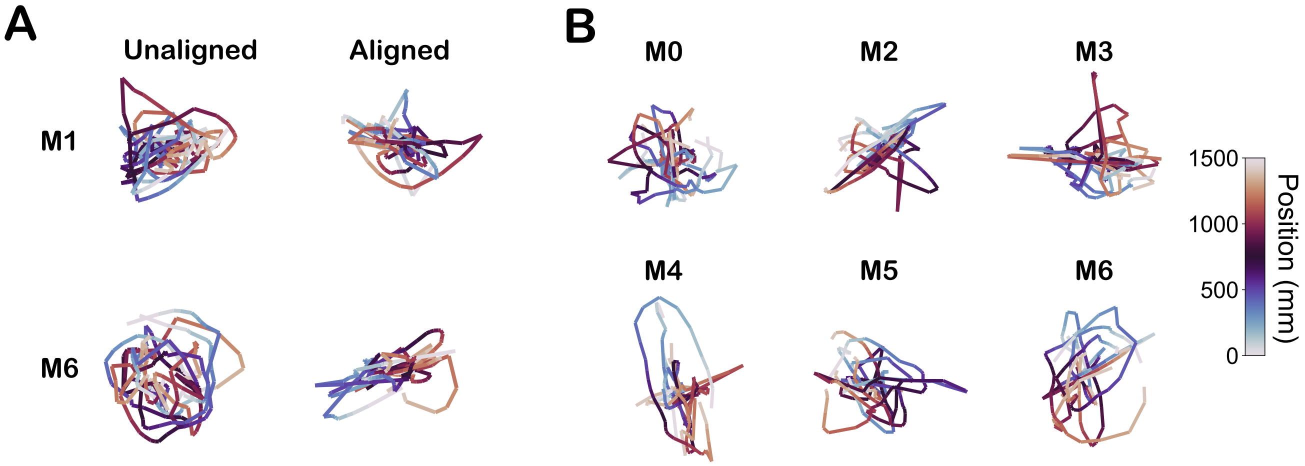

Fig. S2. Alignment controls. A, Each row shows the trial-average PCA of each session before and after alignment for "shifted" recordings. Each session has been shifted by a random amount, and then aligned using CCA. Since neuronal activity has a different encoding with position for every session, the alignment process fails. This is shown for two mice (M1 and M6) and one random selection of session shifts for each mouse. **B,** Trial-averaged PCAs of each session after alignment for "shifted" recordings, for each remaining mice. As in the previous panel, this shows a single random selection of session shifts for each mouse.

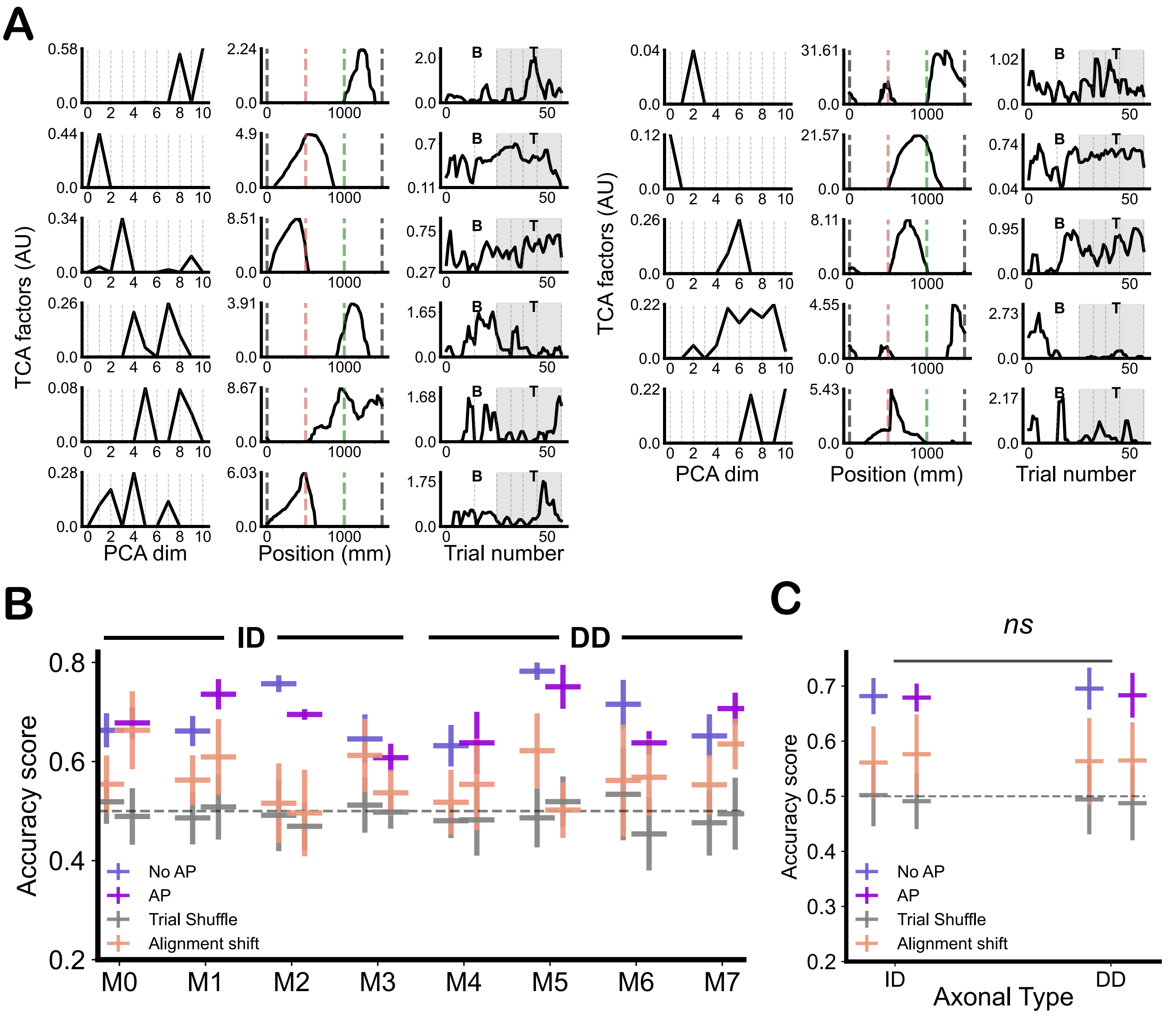

Fig. S3. Air puff decoding accuracy. A, TCA factors for mouse 6 (DD). As many factors as dimensions needed to explain at least 90% of the variance on each session were chosen, in this case $N_{TCA} = 11$. Left column shows the PCA factors. middle column positions factors with dashed lines indicating the reward (gray), air puff (red), and last belt transition (green) locations. Third column shows the trial factors for baseline (B) and training (T) trials. Trials where there air puff is present are colored in gray. **B,** Mouse-averaged raw accuracy scores (proportion of correct labels) for each mouse and class. Dashed line represents the expected chance of random guessing. Vertical bars represent the standard deviation of the data. **C,** Axonal-type-averaged raw accuracy scores (proportion of correct labels) for each mouse type and class. Dashed line represents the expected chance of random guessing. Vertical bars represent the standard deviation of the data.

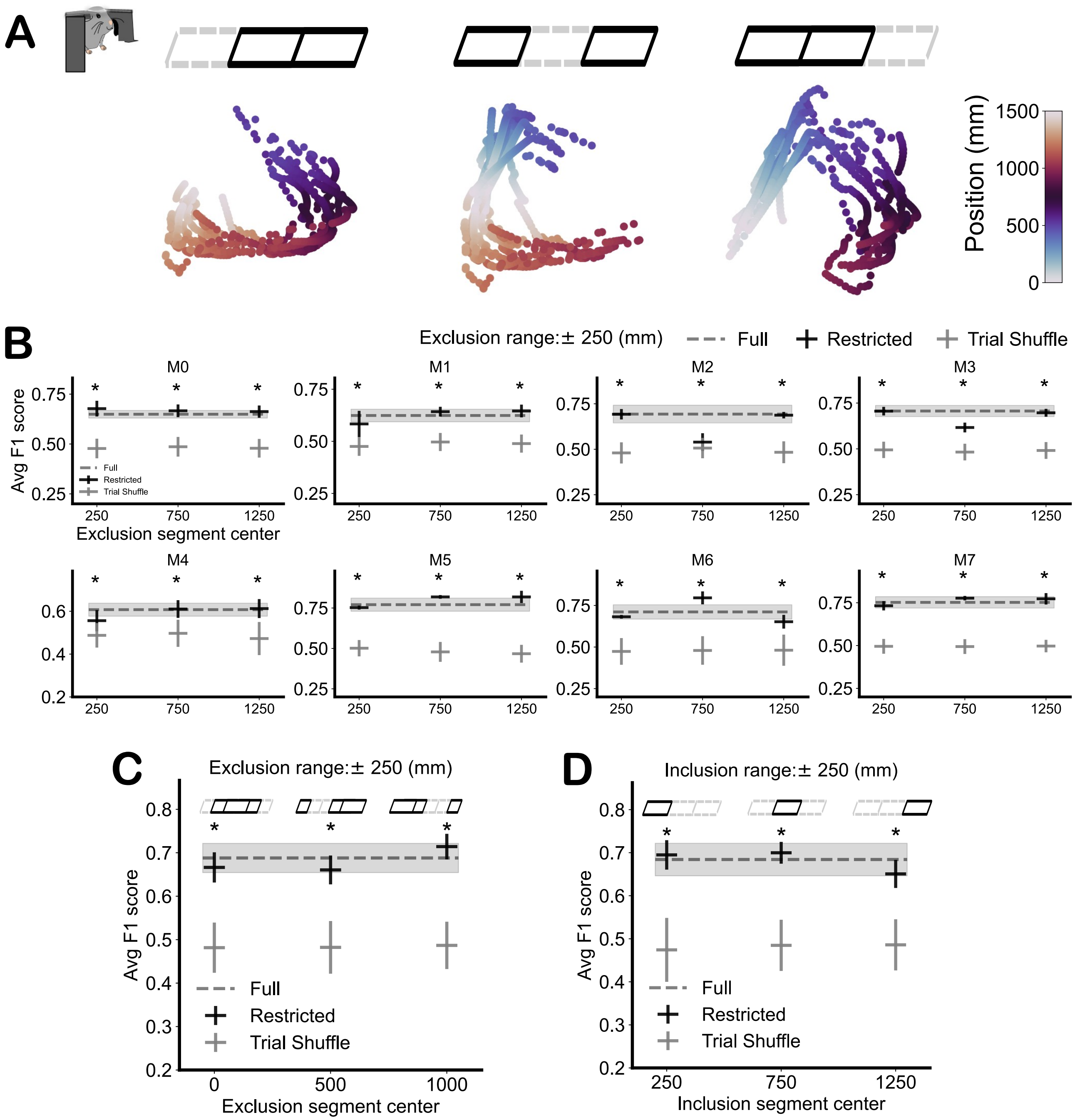

Fig. S4. Excluding sections of the belt does not change air puff decodability results. **A,** Example PCAs from mouse 6 after excluding one of three sections fo the periodic belt, colored according to position. The top drawings show which third of the belt was excluded. **B,** F1 scores after excluding one third of the belt for each mouse. The dashed line and the gray area show the average F1 scores and their standard deviation, respectively, when no exclusion is performed. Asterisks represent significance with shuffle (Mann-Whitney U test, $p < 0.05$). **C,** Mouse-averaged F1 scores after excluding one third of the belt, but centered around belt section transitions. There was no significant drop in decodability, indicating that the overall results were not affected by the stopping behavior of the animal. **D,** Mouse-averaged F1 scores after excluding two thirds of the belt. Once again there was no significant drop in decodability.

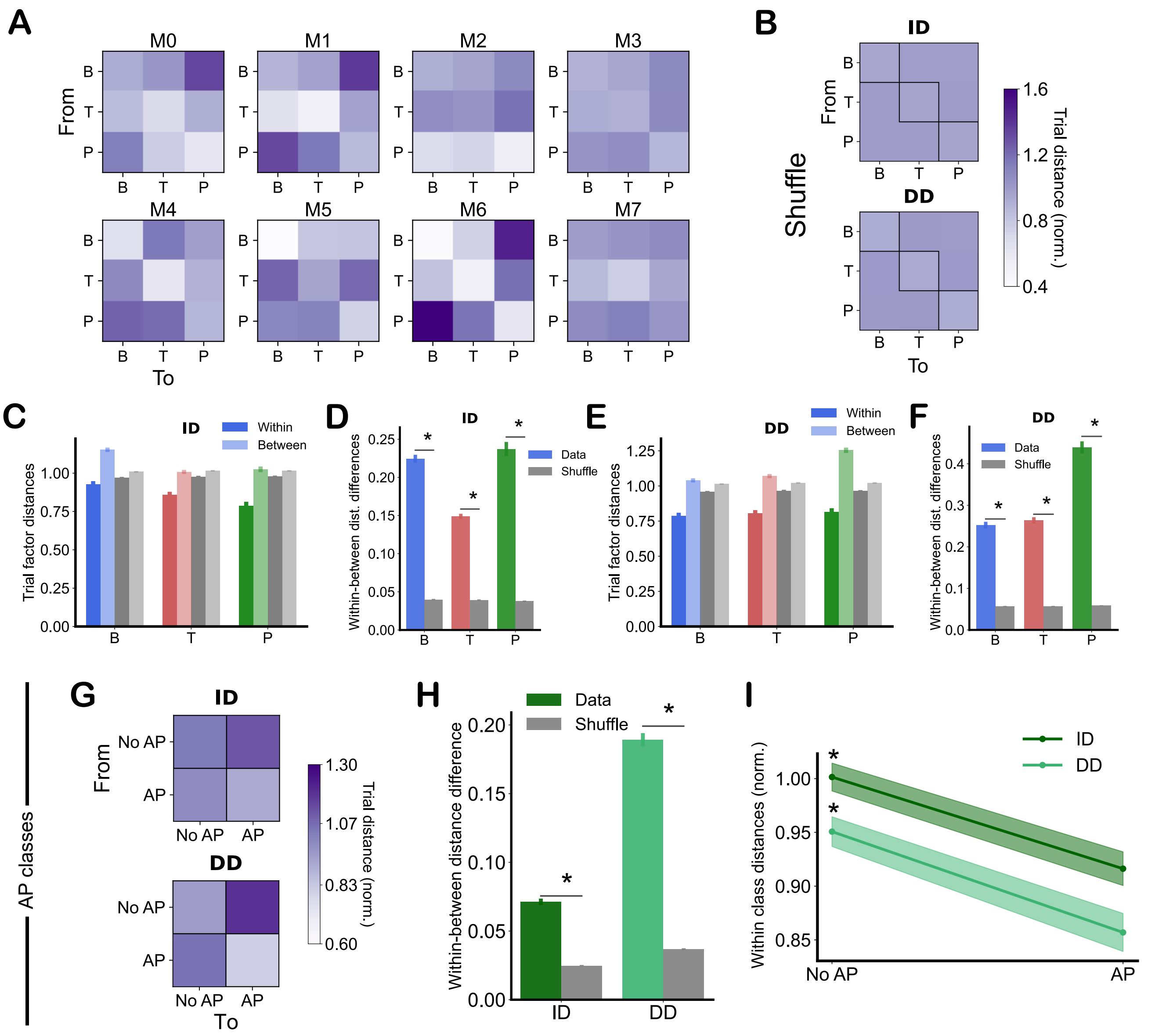

Fig. S5. Euclidean distance calculations for trial factors. A, Colormaps of the average normalized distance from a trial to each centroid, by mouse. The diagonal elements show the average within-cluster distance. **B,** Colormaps of the average normalized distance from a trial to each centroid, by axonal type, after random trial label shuffling. The diagonal elements show the average within-cluster distance. This colormap shows more homogeneity than panel A or Fig. 4C. **C,** Mouse-normalized average distances between a trial factor and the center of mass of its own session type ("within") or the center of masses of the other session types ("between"), for each session type. Gray bars represent the shuffled results. Vertical lines show the standard error of the mean. Results shown for ID axons only. **D,** The "within" and "between" distances from the previous panel are subtracted in order to quantify cluster separability. This panel shows the mouse-normalized average distance differences for each session type. Gray bars represent the shuffled results. Asterisks indicate significant (Mann-Whitney U test, $p < 0.05$). Here, results shown for ID axons only. **E,F,** Same as panels C,D but for DD axons. **G,** Same as panel A, but this time grouping trials by air puff presence (T sessions, "AP") and no air puff presence (B and P sessions, "NoAP"). **H,** Average difference between the "within" and "between" normalized distances from panel G, for each axonal type, for both the original data and the label-shuffle. Shaded areas represent the standard error of the data. Significance (Mann-Whitney U test, $p < 0.05$) was computed with respect to label-shuffle. **I,** Average distances within a cluster for AP and NoAP classes. Asterisk indicates significance (Mann-Whitney U test, $p < 0.05$) between the two classes.

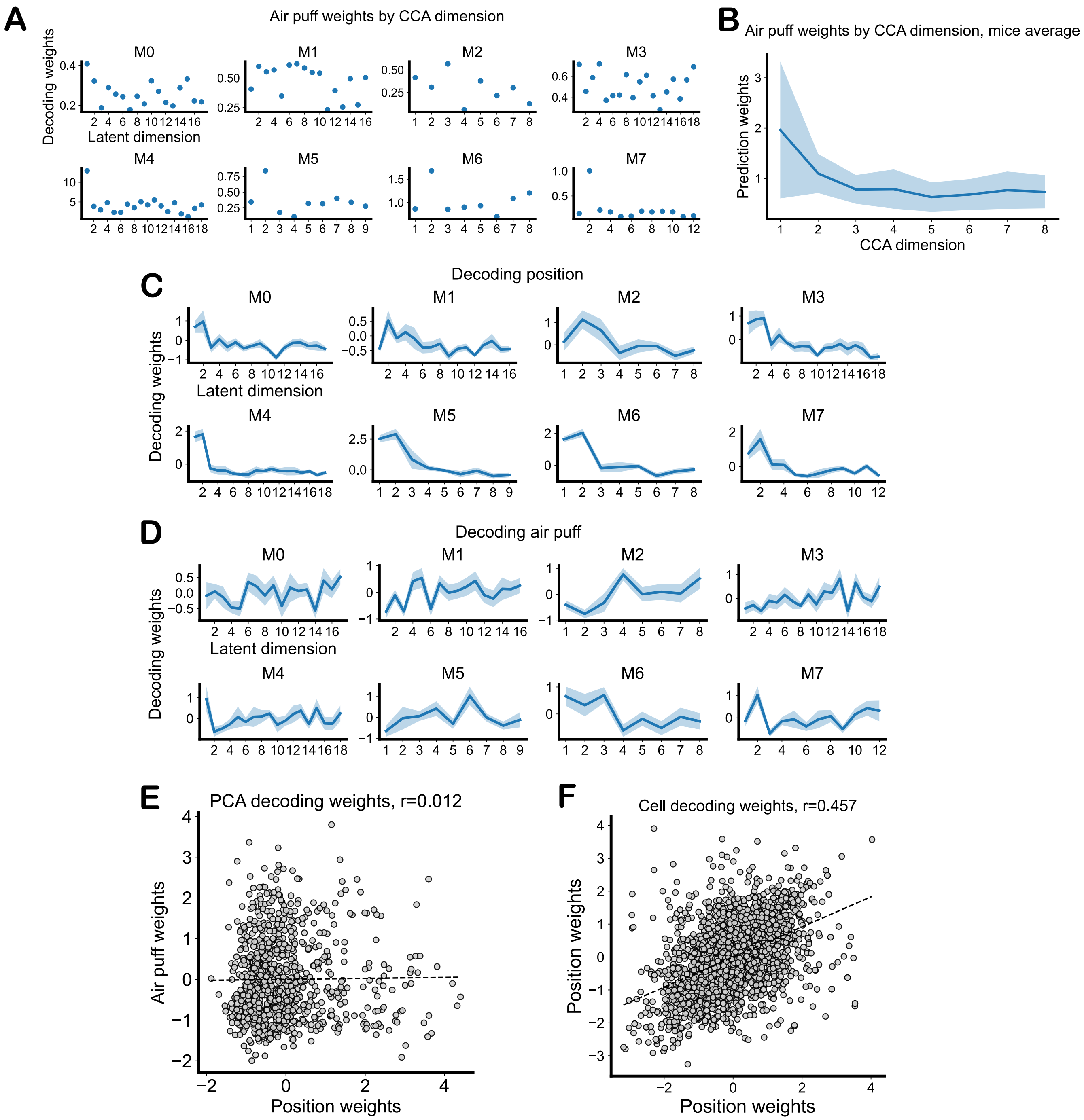

Fig. S6. Decoding weights for position and air puff. A, Air puff decoding weights for each aligned CCA dimension and mouse. **B,** Average normalized air puff decoding weights by CCA latent dimension across mice. Shaded area represents the standard error of the mean. **C,** Average, session-normalized position decoding weights by PCA dimension for each mouse. Shaded area represents the standard error of the mean. **D,** Same as C, but for air puff decoding weights. **E** Plotting air puff decoding weights against position decoding weights for each PCA dimension. Dashed line shows linear regression, which was non-significant with a p-value of 0.28. **F** Position decoding weights computed from two different subsets of the data, plotted against each other. We considered two thirds for each calculation, allowing for some overlap. Linear regression coefficient shown on top, which was significant (p-value under 0.05).

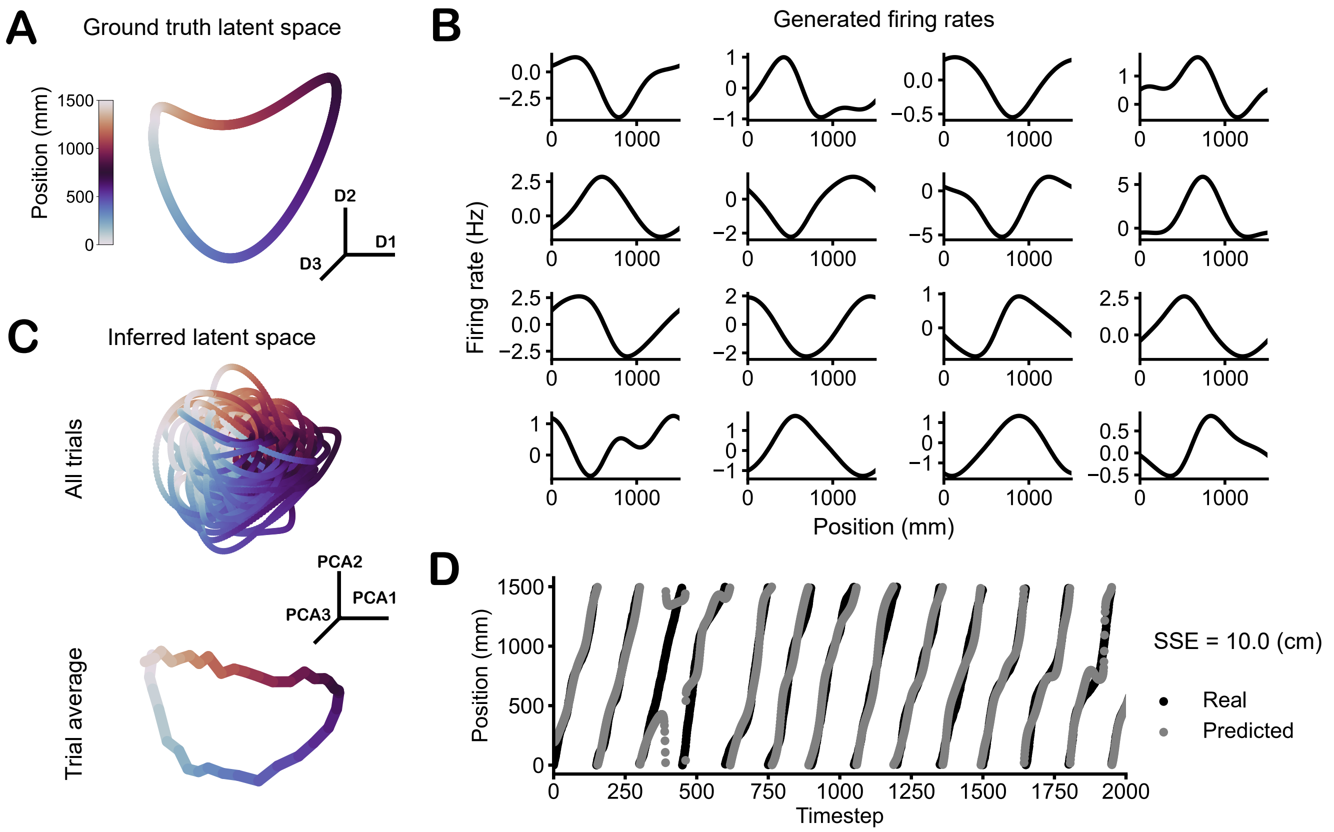

Fig. S7. Generating simulated data using a low-dimensional latent space. A, Example of a three dimensional latent space used as the ground truth to generate the simulate data. **B,** Sixteen firing rate functions randomly generated from the latent space in A. **C,** Inferred latent space from a generated session using PCA. All trials are shown at the top, and the trial average on the bottom. Note that the ground truth shape from A is not fully recovered. **D,** Predicting simulated positions from generated data using support vector regression. The sum of squares error is shown on the right.

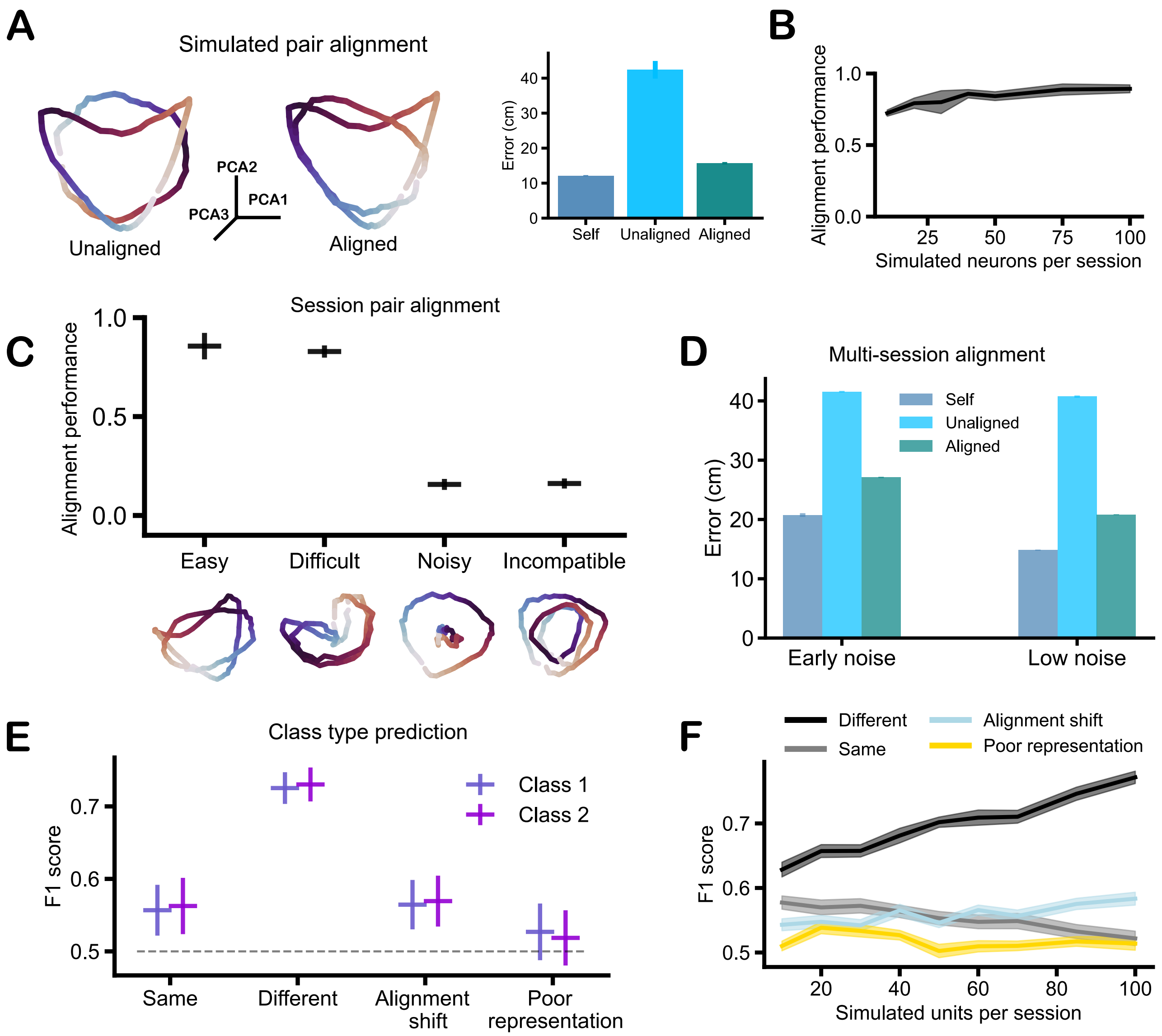

Fig. S8. Analyzing alignment and trial type decoding in simulated data. A, Trial-averaged PCAs of simulated data with similar ground truth, before (A, left) and after (A, middle) alignment using CCA, show low cross-session prediction errors after alignment (A, right). **B,** Alignment performance for the "Easy" session pair type, as a function of the number of neurons. **C,** Alignment performance for various kinds of simulated session pairs, which quantifies how close the aligned error is to the "self" error, relative to unaligned. Examples of aligned PCAs are shown at the bottom for session pairs whose ground truth latent spaces are similar ("Easy"), different in complicated, non-linear ways ("Difficult"), have the second session represent gaussian noise ("Noisy"), or a different task ("Incompatible") (See Methods for more details on each type). Alignment performance is shown only for the first session of the pair. **D,** Results of aligning multiple (nine) simulated sessions using mCCA, with initially noisier sessions ("Early noise") or constant low noise ("Low noise"). This panel is analogous to Fig. 2F. **E,** F1 scores for decoding the trials of two types of generated sessions, analogous to air puff detection. 3 sessions of each class were simulated, aligned, and decoded on a trial-by-trial basis using trial factors from TCA. This was done for cases where the ground truth latent spaces of the two session types were the same ("Same"), different but still constituted a good space representation ("Different"), were randomly shifted before alignment ("Alignment shift"), or were too noisy ("Poor representation"). **F,** F1 scores for the session types from E, as a function of the number of neurons. **All,** Standard error of the mean is shown as either a vertical line (A, C, D, E) or a shaded area (B, F).
